# Expanding the application of chlorinated anilines as molecular templates to achieve a series of solid-state [2 + 2] cycloaddition reactions

**DOI:** 10.3389/fchem.2025.1698626

**Published:** 2025-12-04

**Authors:** Grace K. White, Daniel K. Unruh, Ryan H. Groeneman

**Affiliations:** 1 Department of Natural Sciences and Mathematics, Webster University, St. Louis, MO, United States; 2 Office of the Vice President for Research, University of Iowa, Iowa City, IA, United States

**Keywords:** co-crystal, organic solid state, [2 + 2] cycloaddition reaction, hydrogen bonding, co-crystal former

## Abstract

The ability to achieve a series of solid-state [2 + 2] cycloaddition reactions within related hydrogen-bonded co-crystals is reported. These multicomponent molecular solids contain either *trans*-1,2-bis(3-pyridyl)ethylene (**3,3-BPE**) or *trans*-1,2-bis(2-pyridyl)ethylene (**2,2-BPE**) as the reactant, along with one of two chlorinated anilines that behave as a template, namely 2,3,5,6-tetrachloroaniline (**C**_**6**_ **H**_**3**_ **Cl**_**4**_ **N**) or 2,4,6-trichloroaniline (**C**_**6**_ **H**_**4**_ **Cl**_**3**_ **N**). For each of the four unique organic solids, the co-crystallization process yields a three-component hydrogen-bonded assembly with a formula of either 2(**C**_**6**_ **H**_**3**_ **Cl**_**4**_ **N**)·(**3,3-BPE**), 2(**C**_**6**_ **H**_**4**_ **Cl**_**3**_ **N**)·(**3,3-BPE**), 2(**C**_**6**_ **H**_**3**_ **Cl**_**4**_ **N**)·(**2,2-BPE**), or 2(**C**_**6**_ **H**_**4**_ **Cl**_**3**_ **N**)·(**2,2-BPE**). In all co-crystals, these anilines template up to a quantitative yield for the photoreaction since they are able to engage in both N-H···N hydrogen bonds and homogeneous face-to-face π–π stacking interactions, which position the ethylene groups within the different reactant molecules in a suitable location to photoreact. These results complete the series for the remaining symmetric bipyridine-based reactants to undergo a solid-state [2 + 2] cycloaddition reaction utilizing these chlorinated anilines. This work expands and illustrates the potential for these chlorinated anilines to serve as reliable molecular templates that crystal engineers can utilize to control the organic solid state and achieve photoreactions.

## 1 Introduction

The intentional design of organic solids that will undergo the light-induced [2 + 2] cycloaddition reaction continues to be an active area of research within crystal engineering (Li and MacGillivray, 2025). In most cases, the photoreaction is achieved using a template-based approach since most reactants are photostable as a single-component solid (Biradha and Santra, 2013; Gan et al., 2018; Kole and Mir, 2022). The template (*i.e*., co-crystal former) overcomes issues with crystal packing by taking advantage of the strength and directionality of non-covalent interactions that yield a different crystal form compared to the single-component structure (MacGillivray, 2008). In general, hydrogen and halogen bonds have been utilized to position a pair of carbon–carbon double bonds (C=C) in a suitable orientation and distance to photoreact and form a cyclobutane-based product (Schmidt, 1971).

**SCHEME 1:**
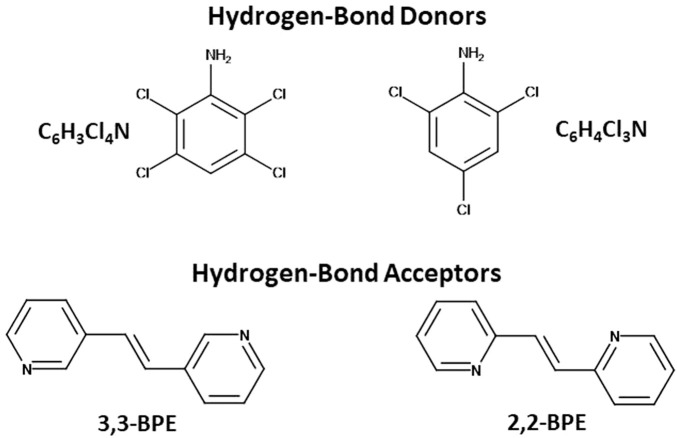
Structures of the hydrogen-bond donors and acceptors within the various co-crystals.

A continued focus for this research group has been the formation of photoreactive co-crystals by exploiting homogeneous face-to-face π–π stacking interactions from various chlorinated aromatics that behave as a molecular template (Andren et al., 2024; Bosch et al., 2023; Shapiro et al., 2021). Recently, we reported the ability of a pair of chlorinated anilines, namely 2,3,5,6-tetrachloroaniline (**C**_**6**_ **H**_**3**_ **Cl**_**4**_ **N**) and 2,4,6-trichloroaniline (**C**_**6**_ **H**_**4**_ **Cl**_**3**_ **N**) (Scheme 1), that behave as templates to achieve a photoreaction when combined with *trans*-1,2-bis(4-pyridyl)ethylene (**4,4-BPE**) (White et al., 2025). These three-component co-crystals were photoreactive since the anilines engaged in both N-H···N hydrogen bonds and π–π stacks in a homogeneous face-to-face pattern that positioned the ethylene group on the reactant in an appropriate location to photoreact. In this original contribution, density functional theory calculations determined that the homogeneous stacking pattern is preferred over a theoretical heterogeneous pattern, which is essential to achieve a photoreactive solid since the reactant was also found in an infinite and homogeneous column, which then satisfied the requirements for a [2 + 2] cycloaddition reaction (White et al., 2025).

**SCHEME 2:**
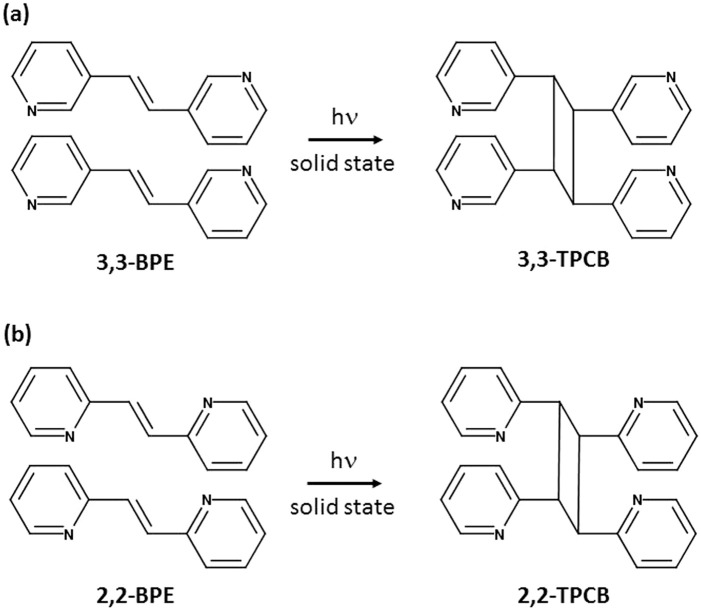
Structure of the solid-state [2 + 2] cycloaddition reaction of **(a) 3,3-BPE** to produce **3,3-TPCB** and **(b) 2,2-BPE** to produce **2,2-TPCB**.

With the goal of expanding this research to the remaining symmetric bipyridine-based reactants, namely *trans*-1,2-bis(3-pyridyl)ethylene (**3,3-BPE**) and *trans*-1,2-bis(2-pyridyl)ethylene (**2,2-BPE**) (Scheme 1), a series of co-crystallization and photochemical studies were performed. The combination of these reactants and the two previously studied chlorinated anilines yielded four additional co-crystals, namely 2(**C**_**6**_ **H**_**3**_ **Cl**_**4**_ **N**)·(**3,3-BPE**), 2(**C**_**6**_ **H**_**4**_ **Cl**_**3**_ **N**)·(**3,3-BPE**), 2(**C**_**6**_ **H**_**3**_ **Cl**_**4**_ **N**)·(**2,2-BPE**), and 2(**C**_**6**_ **H**_**4**_ **Cl**_**3**_ **N**)·(**2,2-BPE**). In all cases, a hydrogen-bonded three-component co-crystal was obtained, where again the chlorinated anilines engage in both N-H···N hydrogen bonds and homogeneous face-to-face π–π stacking interactions that position the ethylene group to undergo a photoreaction upon exposure to ultraviolet light. In three of the four co-crystals, a quantitative photoreaction was achieved, generating a stereoselective photoproduct, either *rctt*-tetrakis(3-pyridyl)cyclobutane (**3,3-TPCB**) (Scheme 2a) or *rctt*-tetrakis(2-pyridyl)cyclobutane (**2,2-TPCB**) (Scheme 2b). The results presented in this study illustrate that these chlorinated anilines are an emerging class of molecular templates to achieve various solid-state [2 + 2] cycloaddition reactions. Finally, taking advantage of the strong tendencies of these chlorinated anilines, along with other functionalized chlorinated benzenes, to stack in infinite and homogeneous arrays that engage in various non-covalent interactions illustrates their great potential as an unexplored co-crystal former for controlling the physical and chemical properties of organic solids.

## 2 Experimental section

### 2.1 Materials

The templates 2,3,5,6-tetrachloroaniline (**C**_**6**_ **H**_**3**_ **Cl**_**4**_ **N**) and 2,4,6-trichloroaniline (**C**_**6**_ **H**_**4**_ **Cl**_**3**_ **N**) along with the reactant *trans*-1,2-bis(2-pyridyl)ethylene (**2,2-BPE**) were all purchased from Sigma-Aldrich Chemical (St. Louis, MO, United States) and used as received. The reactant *trans*-1,2-bis(3-pyridyl)ethylene (**3,3-BPE**) was prepared using a previously reported method (Gordillo et al., 2013; Quentin and MacGillivray, 2020a; 2020b). Reagent-grade ethanol was also purchased from Sigma-Aldrich Chemical and was used without purification. All co-crystallization studies were performed in 20 mL scintillation vials.

### 2.2 General methods

Photoreactions were conducted using UV-radiation from a 450 W medium-pressure mercury lamp in an ACE glass photochemistry cabinet. Each co-crystal was dried and placed between a pair of Pyrex glass plates for irradiation. Then, all of the glass-plated co-crystal samples were placed into the photoreactor and exposed to broadband ultraviolet light from the mercury vapor bulb. All of the co-crystal solids were mixed daily to expose a fresh surface to ultraviolet light. The photoreactivity of each co-crystal was determined using ^1^H nuclear magnetic resonance spectroscopy (^1^H NMR) on a Bruker Ascend Evo 400 MHz Spectrometer using DMSO-*d*_*6*_ as the solvent. Single-crystal X-ray diffraction data were collected on a Bruker D8 VENTURE DUO diffractometer equipped with an IµS 3.0 microfocus source operated at 75 W (50 kV, 1.5 mA) to generate Mo Kα radiation (λ = 0.71073 Å) using a PHOTON III detector. Powder X-ray diffraction data were collected at room temperature on a Bruker D8 Advance X-ray Diffractometer using Cu K_α_ radiation (λ = 1.54056 Å) between 5° and 40° two-theta.

### 2.3 Synthesis of co-crystals that contain *trans*-1,2-bis(3-pyridyl)ethylene

Co-crystals of 2(**C**_**6**_ **H**_**3**_ **Cl**_**4**_ **N**)·(**3,3-BPE**) and 2(**C**_**6**_ **H**_**4**_ **Cl**_**3**_ **N**)·(**3,3-BPE**) were both prepared by dissolving 25.0 mg of **3,3-BPE** in 2.0 mL of ethanol, which was then combined with a separate 1.0 mL ethanol solution containing either 63.4 mg of **C**_**6**_ **H**_**3**_ **Cl**_**4**_ **N** or 53.9 mg of **C**_**6**_ **H**_**4**_ **Cl**_**3**_ **N** (1:2 molar ratio). The caps were removed from the combined solutions to allow for slow evaporation. Within 2 days, along with the loss of most solvent, crystals formed that were suitable for single-crystal and powder X-ray diffraction experiments.

### 2.4 Synthesis of co-crystals that contain *trans*-1,2-bis(2-pyridyl)ethylene

In a similar manner, co-crystals of 2(**C**_**6**_ **H**_**3**_ **Cl**_**4**_ **N**)·(**2,2-BPE**) and 2(**C**_**6**_ **H**_**4**_ **Cl**_**3**_ **N**)·(**2,2-BPE**) were prepared by dissolving 25.0 mg of **2,2-BPE** in 2.0 mL of ethanol; then, it was combined with either a 1.0 mL ethanol solution of 63.4 mg of **C**_**6**_ **H**_**3**_ **Cl**_**4**_ **N** or 53.9 mg of **C**_**6**_ **H**_**4**_ **Cl**_**3**_ **N** (1:2 molar ratio). After combining the two solutions, the cap was removed to allow for slow evaporation. Again, within 2 days, crystals formed that were suitable for single-crystal and powder X-ray diffraction experiments after the loss of most of the ethanol.

## 3 Results and discussion

### 3.1 Structure and photoreactivity of 2(C_6_H_3_Cl_4_N)·(3,3-BPE)

**FIGURE 1:**
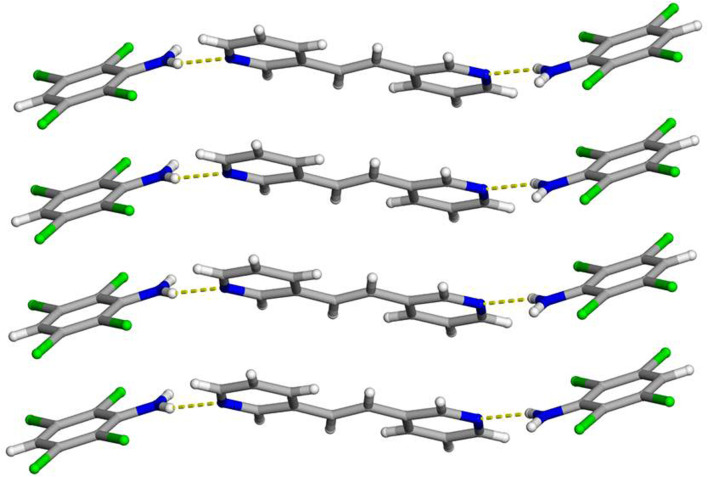
X-ray crystal structure of 2(**C**_**6**_ **H**_**3**_ **Cl**_**4**_ **N**)·(**3,3-BPE**) illustrating the N-H···N hydrogen bonds along with the infinite homogeneous face-to-face π–π stacking pattern of the aromatic rings. The N-H···N hydrogen bonds are shown as yellow dashed lines.

**FIGURE 2:**
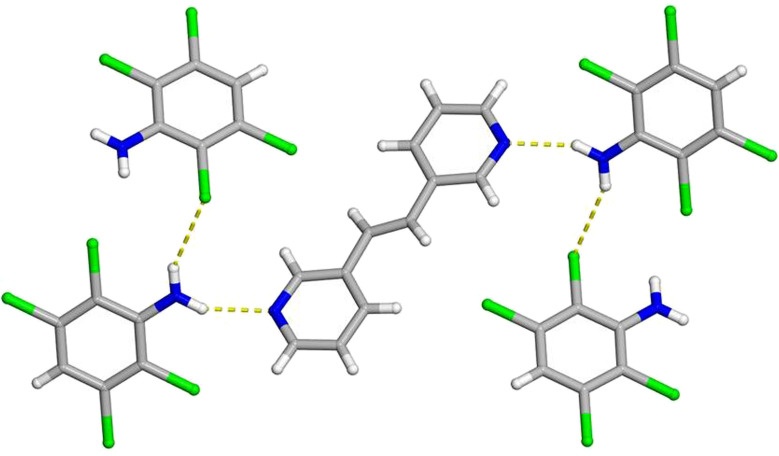
X-ray crystal structure of 2(**C**_**6**_ **H**_**3**_ **Cl**_**4**_ **N**)·(**3,3-BPE**) illustrating the N-H···N hydrogen bonds along with the N-H···Cl contacts. The N-H···N hydrogen bonds and N-H···Cl contacts are shown as yellow dashed lines.

**FIGURE 3:**
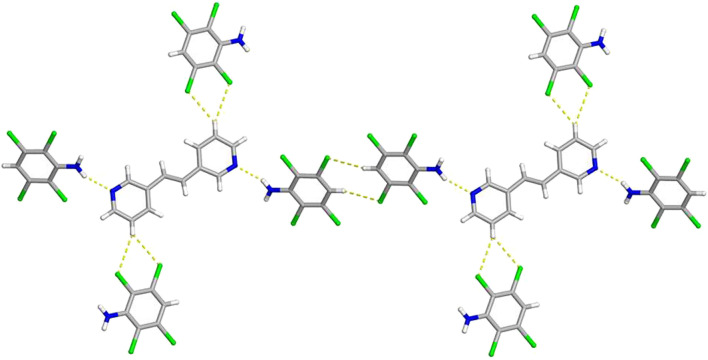
X-ray crystal structure of 2(**C**_**6**_ **H**_**3**_ **Cl**_**4**_ **N**)·(**3,3-BPE**) illustrating the N-H···N hydrogen bonds and the different types of C-H···Cl contacts. The N-H···N hydrogen bonds and C-H···Cl contacts are shown as yellow dashed lines.

The molecular components of 2(**C**_**6**_ **H**_**3**_ **Cl**_**4**_ **N**)·(**3,3-BPE**) crystallize into the centrosymmetric monoclinic space group *C*2/*c*. Within the asymmetric unit is a whole molecule of **C**_**6**_ **H**_**3**_ **Cl**_**4**_ **N** and half a molecule of **3,3-BPE** where applying inversion symmetry generates the remainder of the reactant molecule. This three-component co-crystal is sustained primarily by N-H···N hydrogen bonds [N···N 2.997 (4) Å] that result in a discrete molecular solid (Figure 1). The second N-H group is found to engage in N-H···Cl contacts [N···Cl 3.619 (5) Å] with an *ortho*-chlorine, with respect to the amine group, on a neighboring aniline (Figure 2). The ethylene bridge within **3,3-BPE** is found to be completely ordered at 290 K. The hydrogen-bond donor and acceptor are found twisted away from each other at a value of 42.60° within the three-component assembly at 290 K (Figure 1). Finally, these three-component hydrogen-bonded assemblies also interact with neighbors through various C-H···Cl contacts (Hathwar et al., 2010), namely linear [C···Cl 3.945 (4) Å] and bifurcated [C···Cl 3.679 (5) and 3.900 (5) Å], which generate an extended solid (Figure 3).

As expected, molecules of **C**_**6**_ **H**_**3**_ **Cl**_**4**_ **N** are found to engage in homogenous face-to-face π–π stacking interactions (Figure 1). These stacked anilines produce an infinite column with a centroid-to-centroid distance of 3.8601 (7) Å, which is equal to the crystallographic *b*-axis. Due to the π-stacking pattern and N-H···N hydrogen bonds, the reactant **3,3-BPE**, along with the ethylene group, is also found in an infinite stack (Figure 1). As a result of translational symmetry, these reactive centers are parallel and within the distance limit to achieve a solid-state [2 + 2] cycloaddition reaction as defined by the topochemical postulate (Schmidt, 1971).

To determine whether 2(**C**_**6**_ **H**_**3**_ **Cl**_**4**_ **N**)·(**3,3-BPE**) would undergo a photoreaction, a dried powdered sample was exposed to ultraviolet radiation in a photochemical cabinet from a mercury vapor bulb. A [2 + 2] cycloaddition reaction was observed using ^1^H NMR by the substantial loss of the olefinic signal at 7.44 ppm on **3,3-BPE,** along with the concomitant appearance of a cyclobutane signal at 4.71 ppm, confirming the formation of the stereospecific photoproduct **3,3-TPCB** (Scheme 2a) (Quentin and MacGillivray, 2020a). An overall yield of 98% for the solid-state [2 + 2] cycloaddition reaction was reached within 60 h of exposure (Supplementary Figures S1, S2).

To determine the purity of the bulk solid and compare it with the single-crystal structure for 2(**C**_**6**_ **H**_**3**_ **Cl**_**4**_ **N**)·(**3,3-BPE**), a powder X-ray diffraction (PXRD) experiment was performed on the resulting solid. The diffractogram confirms that the solid material matches the reported co-crystal structure based on its calculated powder pattern from the single-crystal data (Supplementary Figure S9). This high level of purity for the bulk supports the observed near-quantitative yield for the photoreaction, since nearly all of the reactant molecules are in a suitable position to photoreact.

### 3.2 Structure and photoreactivity of 2(C_6_H_4_Cl_3_N)·(3,3-BPE)

**FIGURE 4:**
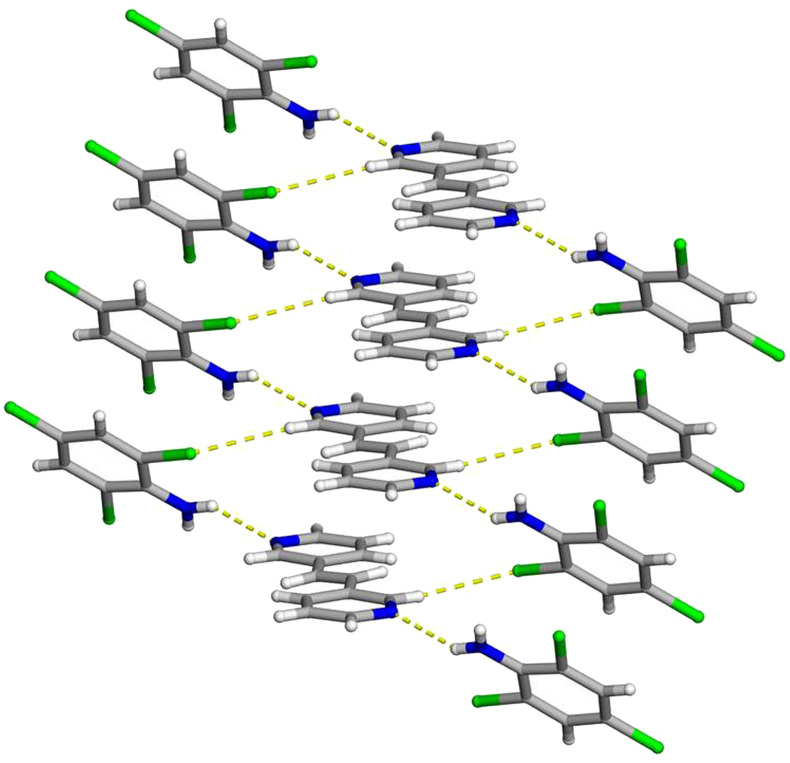
X-ray crystal structure of 2(**C**_**6**_ **H**_**4**_ **Cl**_**3**_ **N**)·(**3,3-BPE**) illustrating the N-H···N hydrogen bonds along with the infinite homogenous face-to-face π–π stacking pattern of the aromatic rings. The N-H···N hydrogen bonds and C-H···Cl contacts are shown as yellow dashed lines.

The co-crystal 2(**C**_**6**_ **H**_**4**_ **Cl**_**3**_ **N**)·(**3,3-BPE**) crystallizes into the centrosymmetric triclinic space group *P*ī. Similar to before, a whole molecule of **C**_**6**_ **H**_**4**_ **Cl**_**3**_ **N**, along with half a molecule of **3,3-BPE**, is found in the asymmetric unit. Applying inversion symmetry generates the remainder of the acceptor molecule and the three-component hydrogen-bonded co-crystal. This discrete molecular solid is sustained predominantly by N-H···N hydrogen bonds [N···N 3.093 (4) Å] (Figure 4). Unlike 2(**C**_**6**_ **H**_**3**_ **Cl**_**4**_ **N**)·(**3,3-BPE**), the second N-H group in 2(**C**_**6**_ **H**_**4**_ **Cl**_**3**_ **N**)·(**3,3-BPE**) does not form a hydrogen bond with any suitable acceptor group. Again, the ethylene group within **3,3-BPE** is completely ordered at 290 K. The hydrogen-bond donor and acceptor are found twisted away from each other within the three-component assembly with a value of 49.06° at 290 K (Figure 4). Neighboring and stacked hydrogen-bonded assemblies are engaged in C-H···Cl contacts [C···Cl 3.736 (3) Å] between the *ortho*-chlorine, with respect to the amine, and the hydrogen atom between the nitrogen and ethylene bridge on the pyridine ring (Figure 4). A similar C-H···Cl contact pattern was also observed within the co-crystal 2(**C**_**6**_ **H**_**4**_ **Cl**_**3**_ **N**)·(**4,4-BPE**) as previously reported by this research group (White et al., 2025).

Essential to achieving a photoreactive co-crystal, both the hydrogen-bond donor and acceptor are once again found in a homogeneous face-to-face π-stacked pattern (Figure 4). These stacked aromatic rings lie along the crystallographic *a*-axis with a centroid-to-centroid distance of 3.890 (1) Å. Due to crystal symmetry, the ethylene groups, within the infinite column, are again found parallel and within a suitable distance to undergo a solid-state photoreaction. As previously stated, the combination of the homogeneous face-to-face π–π stacking pattern along with N-H···N hydrogen bonds positions the reactive centers in an appropriate location to undergo a [2 + 2] cycloaddition reaction.

To determine the photoreactivity of 2(**C**_**6**_ **H**_**4**_ **Cl**_**3**_ **N**)·(**3,3-BPE**), a dried powdered sample was placed into a photoreactor and exposed to ultraviolet radiation. A [2 + 2] cycloaddition reaction was confirmed by the complete loss of the olefinic signal at 7.44 ppm on **3,3-BPE**, along with the concomitant appearance of a cyclobutane signal associated with **3,3-TPCB** at 4.71 ppm in the ^1^H NMR spectra (Scheme 2a) (Quentin and MacGillivray, 2020a). The yield for the [2 + 2] cycloaddition reaction reaches a quantitative level after only 16 h of exposure to ultraviolet light (Supplementary Figures S3, S4).

The structure of the bulk solid containing 2(**C**_**6**_ **H**_**4**_ **Cl**_**3**_ **N**)·(**3,3-BPE**) was investigated using a PXRD experiment. Comparing the observed diffractogram to the theoretical powder pattern for the single-crystal structure of 2(**C**_**6**_ **H**_**4**_ **Cl**_**3**_ **N**)·(**3,3-BPE**) confirms a high level of purity for the resulting solid (Supplementary Figure S11). This good agreement of the diffraction peaks supports the quantitative yield of the photoreaction due to the high purity of the bulk material.

### 3.3 Structure and photoreactivity of 2(C_6_H_3_Cl_4_N)·(2,2-BPE)

**FIGURE 5:**
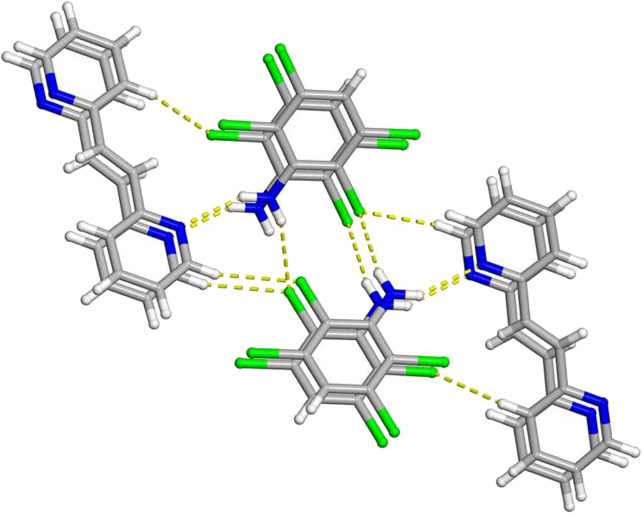
X-ray crystal structure of 2(**C**_**6**_ **H**_**3**_ **Cl**_**4**_ **N**)·(**2,2-BPE**) illustrating the N-H···N hydrogen bonds along with the N-H···Cl and C-H···Cl contacts. The N-H···N hydrogen bonds, N-H···Cl contacts, and C-H···Cl contacts are shown as yellow dashed lines.

**FIGURE 6:**
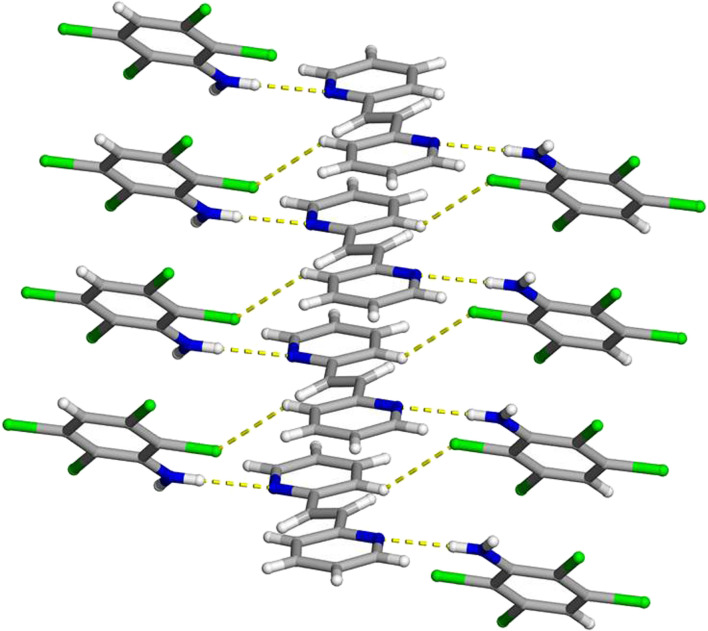
X-ray crystal structure of 2(**C**_**6**_ **H**_**3**_ **Cl**_**4**_ **N**)·(**2,2-BPE**) illustrating the N-H···N hydrogen bonds along with the infinite homogenous face-to-face π–π stacking pattern of the aromatic rings. The N-H···N hydrogen bonds and C-H···Cl contacts are shown as yellow dashed lines.

**FIGURE 7:**
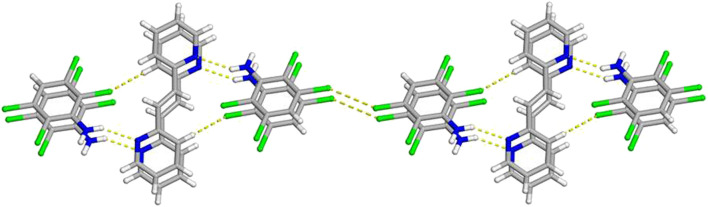
X-ray crystal structure of 2(**C**_**6**_ **H**_**3**_ **Cl**_**4**_ **N**)·(**2,2-BPE**) illustrating the Type I Cl···Cl interactions between neighboring three-component hydrogen-bonded assemblies. The N-H···N hydrogen bonds, C-H···Cl contacts, and type I Cl···Cl interactions are shown as yellow dashed lines.

Single-crystal diffraction data revealed that the molecules in 2(**C**_**6**_ **H**_**3**_ **Cl**_**4**_ **N**)·(**2,2-BPE**) crystallize in the centrosymmetric monoclinic space group *P*2_1_/*n*. Within the asymmetric unit is a whole molecule of **C**_**6**_ **H**_**3**_ **Cl**_**4**_ **N** along with half a molecule of **2,2-BPE** where again inversion symmetry generates the remainder of the reactant molecule. The co-crystal 2(**C**_**6**_ **H**_**3**_ **Cl**_**4**_ **N**)·(**2,2-BPE**) is held together primarily by N-H···N hydrogen bonds [N···N 3.076 (2) Å] that result in a discrete three-component hydrogen-bonded solid (Figures 5, 6). The second unique N-H group interacts with an adjacent donor via N-H···Cl contacts [N···Cl 3.677 (2) Å] to an *ortho*-chlorine with respect to the amine group (Figures 5, 6). The ethylene group within **2,2-BPE** is found to be ordered at 290 K within 2(**C**_**6**_ **H**_**3**_ **Cl**_**4**_ **N**)·(**2,2-BPE**). The hydrogen-bond donor and acceptor are twisted at 47.36° from coplanar within the discrete assembly at 290 K (Figure 6). Nearest hydrogen-bonded arrays interact via C-H···Cl contacts [C···Cl 3.709 (2) Å] between an *ortho*-chlorine to the amine group and an *ortho*-hydrogen to the ethylene bridge (Figures 5, 6). Finally, these assemblies also interact via Type I Cl···Cl interactions [Cl···Cl 3.490 (1) Å] between a pair of *meta*-chlorines to the amine group (Figure 7) (Mukherjee et al., 2014). The combination of all of these non-covalent interactions results in a three-dimensional extended structure.

Paramount to the formation of a photoreactive co-crystal are the homogeneous face-to-face π–π stacking interactions of both the hydrogen-bond donor and acceptor within 2(**C**_**6**_ **H**_**3**_ **Cl**_**4**_ **N**)·(**2,2-BPE**) (Figures 5, 6). These stacked aromatics result in a series of infinite columns of the template and reactant molecules. These aromatic rings, along with the ethylene group, are separated by a centroid-to-centroid distance of 3.8918 (4) Å, which is equivalent to the crystallographic *b*-axis. Within these infinite arrays, the ethylene groups are parallel and well within the distance requirement for a solid-state photoreaction due to translational symmetry between unit cells.

To determine whether the co-crystal 2(**C**_**6**_ **H**_**3**_ **Cl**_**4**_ **N**)·(**2,2-BPE**) would be photoreactive, a dried powdered sample was placed in a photochemical cabinet and exposed to ultraviolet light. As expected, a [2 + 2] cycloaddition reaction was observed via ^1^H NMR. This is evident by the loss of the olefinic signal at 7.71 ppm on **2,2-BPE**, along with the simultaneous appearance of a cyclobutane signal at 4.91 ppm, which confirms the formation of the stereospecific photoproduct **2,2-TPCB** (Scheme 2b) (Colmanet et al., 2025; Quentin and MacGillivray, 2020a). This quantitative yield of the cycloaddition reaction was reached within 40 h of exposure to UV light (Supplementary Figures S5, S6).

As before, the resulting bulk solid containing 2(**C**_**6**_ **H**_**3**_ **Cl**_**4**_ **N**)·(**2,2-BPE**) was investigated by PXRD to determine its overall purity. When comparing the observed diffractogram to the theoretical pattern, based upon the single-crystal structure, it is clear that the solid is in good agreement with the co-crystal (Supplementary Figure S10). Similar to before, this high level of purity for the resulting solid supports the observed quantitative yield for the bulk since all of the reactant molecules are in an appropriate site to undergo a photoreaction.

### 3.4 Structure and photoreactivity of 2(C_6_H_4_Cl_3_N)·(2,2-BPE)

**FIGURE 8:**
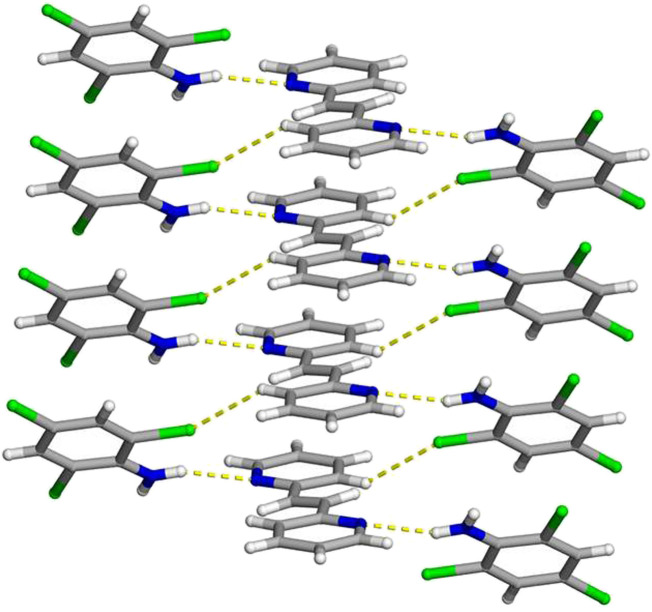
X-ray crystal structure of 2(**C**_**6**_ **H**_**4**_ **Cl**_**3**_ **N**)·(**2,2-BPE**) illustrating the N-H···N hydrogen bonds along with the infinite homogenous face-to-face π–π stacking pattern of the aromatic rings. The N-H···N hydrogen bonds and C-H···Cl contacts are shown as yellow dashed lines.

The molecular components within the co-crystal 2(**C**_**6**_ **H**_**4**_ **Cl**_**3**_ **N**)·(**2,2-BPE**) crystallize in the centrosymmetric triclinic space group *P*ī. As in all the previous co-crystals, a whole hydrogen-bond donor, in this case **C**_**6**_ **H**_**4**_ **Cl**_**3**_ **N**, along with a half of a hydrogen-bond acceptor, namely **2,2-BPE**, are found in the asymmetric unit and applying inversion symmetry generates the remainder of the molecule and the three-component assembly. The co-crystal is sustained by N-H···N hydrogen bonds [N···N 3.057 (3) Å] that result in a discrete molecular solid (Figure 8). Similar to 2(**C**_**6**_ **H**_**4**_ **Cl**_**3**_ **N**)·(**3,3-BPE**), the second N-H group within 2(**C**_**6**_ **H**_**4**_ **Cl**_**3**_ **N**)·(**2,2-BPE**) does not form a hydrogen bond with any suitable acceptor. As observed in all of the previous co-crystals, the ethylene group within the reactant is found to be fully ordered at 290 K. The hydrogen-bonded components in the three-component assembly are rotated from each other by 47.83° at 290 K (Figure 8). The stacked three-component hydrogen-bonded assemblies interact via C-H···Cl contacts [C···Cl 3.682 (2) Å] between the *ortho*-chlorine and *ortho*-hydrogen to the amine group and ethylene bridge, respectively (Figure 8).

Important to achieve a photoreactive co-crystal, the hydrogen-bond donor **C**_**6**_ **H**_**4**_ **Cl**_**3**_ **N**, along with the reactant **2,2-BPE**, is found to be π-stacked in a homogeneous and face-to-face pattern. These stacked aniline and **2,2-BPE** molecules have a centroid-to-centroid distance of 3.8748 (4) Å, which is equal to the crystallographic *a*-axis. The reactive ethylene groups, within the infinite stacks, are again found to be parallel and within a distance that is suitable to undergo a [2 + 2] cycloaddition reaction.

The photoreactivity of 2(**C**_**6**_ **H**_**4**_ **Cl**_**3**_ **N**)·(**2,2-BPE**) was investigated by taking a dried powdered sample and placing it into a photochemical cabinet to be exposed to ultraviolet light. As in previous co-crystals, a [2 + 2] cycloaddition reaction was observed by the complete loss of the olefinic signal at 7.71 ppm on **2,2-BPE**, along with the concomitant appearance of a cylcobutane signal at 4.91 ppm in the ^1^H NMR spectra, which confirms the formation of the stereoselective photoproduct **2,2-TPCB** at (Scheme 2b) (Colmanet et al., 2025; Quentin and MacGillivray, 2020a). A quantitative yield for the [2 + 2] cycloaddition reaction was reached after 60 h of exposure (Supplementary Figures S7, S8). As observed in all the co-crystals in this contribution, the combination of the homogeneous face-to-face π–π stacking pattern, along with the N-H···N hydrogen bonds, places the ethylene groups in an appropriate position to undergo a solid-state photoreaction.

The bulk solid that contained 2(**C**_**6**_ **H**_**4**_ **Cl**_**3**_ **N**)·(**2,2-BPE**) was also investigated using a PXRD experiment. The observed diffractogram of the sample matches the theoretical powder pattern for the single-crystal structure of 2(**C**_**6**_ **H**_**4**_ **Cl**_**3**_ **N**)·(**2,2-BPE**), which confirms a high level of purity for the bulk material (Supplementary Figure S12). This level of agreement between the observed and theoretical powder diffractograms supports the quantitative yield for the photoreaction.

## 4 Conclusion

In this contribution*,* we report the expanded application of **C**_**6**_ **H**_**3**_ **Cl**_**4**_ **N** and **C**_**6**_ **H**_**4**_ **Cl**_**3**_ **N** as molecular templates to achieve solid-state [2 + 2] cycloaddition reactions with the remaining two symmetric bipyridine-based reactants. In all cases, these chlorinated anilines position the ethylene group within the reactant molecule in a suitable location to photoreact due to the combination of N-H···N hydrogen bond and homogeneous face-to-face π–π stacking forces. Currently, we are investigating the catalytic ability of these templates to achieve a high-yielding photoreaction using a substoichiometric amount of the template (Colmanet et al., 2025; Holdaway et al., 2024).

## Data Availability

The datasets presented in this study can be found in online repositories. The names of the repository/repositories and accession number(s) can be found in the article/Supplementary Material.
