# Supplementary material for: Expanding the application of chlorinated anilines as molecular templates to achieve a series of solid-state [2 + 2] cycloaddition reactions
Source: Front Chem. 2025 Dec 4;13:1698626. doi: 10.3389/fchem.2025.1698626 (PMC12711828; doi:10.3389/fchem.2025.1698626)
Supplement: Supplementary file 1 [file DataSheet1.docx]

Supporting Information

for

**Expanding the Application of Chlorinated-anilines as Molecular Templates to Achieve a Series of Solid-State [2 + 2] Cycloaddition Reactions**

Grace K. White, Daniel K. Unruh, and Ryan H. Groeneman^*^

Department of Natural Sciences and Mathematics, Webster University, St. Louis, Missouri, USA

Office of the Vice President for Research, University of Iowa, Iowa City, Iowa, USA

1. Single X-ray Diffraction Information and Data Table S2-S4

2. ^1^H NMR Spectroscopic Data S5-S8

3. Powder X-ray Diffraction Data S9-S12**1. Single X-ray Diffraction Information and Data Table**

Data were collected on a Bruker D8 VENTURE DUO diffractometer equipped with a IµS 3.0 microfocus source operated at 75 W (50 kV, 1.5 mA) to generate Mo Kα radiation (λ = 0.71073 Å) and a PHOTON III detector. Crystals were transferred from the vial and placed on a glass slide in Paratone-N oil. A Zeiss Stemi 305 microscope was used to identify a suitable specimen for X-ray diffraction from a representative sample of the material. The selected crystal and a small amount of the oil were collected on a MiTeGen 100-micron MicroLoop and transferred to the instrument. The sample was optically centered with the aid of a video camera to ensure that no translations were observed as the crystal was rotated through all positions. A unit cell collection was then carried out. After it was determined that the unit cell was not present in the CCDC database a data collection strategy was calculated by *APEX6*.^1^ The crystal was measured for size, morphology, and color.

After data collection, the unit cell was re-determined using a subset of the full data collection. Intensity data were corrected for Lorentz, polarization, and background effects using the *APEX6*.^1^ A numerical absorption correction was applied based on a Gaussian integration over a multifaceted crystal and followed by a semi-empirical correction for adsorption applied using *SADABS*.^1^ The program *SHELXT*^2^ was used for the initial structure solution, and *SHELXL*^3^ was used for refinement of the structure. Both programs were utilized within the OLEX2 software.^4^ Hydrogen atoms bound to carbon atoms were located in the difference Fourier map and were geometrically constrained using the appropriate AFIX commands.

**Table S1**. Single-crystal X-ray diffraction data for 2(**C_6_H_3_Cl_4_N**)·(**3,3-BPE**) and 2(**C_6_H_4_Cl_3_N**)·(**3,3-BPE**) at 290 K.

| compound name | 2(**C_6_H_3_Cl_4_N**)·(**3,3-BPE**) | 2(**C_6_H_4_Cl_3_N**)·(**3,3-BPE**) |
| --- | --- | --- |
| chemical formula | C_24_H_16_Cl_8_N_4_ | C_24_H_18_N_4_Cl_6_ |
| formula mass | 644.01 | 575.12 |
| crystal system | Monoclinic | Triclinic |
| space group | *C*2*/*c | *P*ī |
| *a* (Å) | 28.413(5) | 3.8900(13) |
| *b* (Å) | 3.8601(7) | 12.556(3) |
| *c* (Å) | 27.439(5) | 14.218(4) |
| α (˚) | 90 | 116.199(7) |
| β (˚) | 118.160(5) | 93.065(10) |
| γ (˚) | 90 | 91.904(9) |
| V (Å^3^) | 2653.2(8) | 621.0(3) |
| ρ_calc_ (g cm^-3^) | 1.612 | 1.538 |
| T (K) | 290 | 290 |
| Z | 4 | 1 |
| radiation type | MoKα (λ = 0.71073) | MoKα (λ = 0.71073) |
| absorption coefficient, μ/mm^-1^ | 0.873 | 0.714 |
| no. of reflections measured | 29501 | 12829 |
| no. of independent reflections | 3291 | 2530 |
| R_int_ | 0.0400 | 0.0592 |
| R_1_ (I > 2σ(I)) | 0.0717 | 0.0376 |
| wR(F^2^) (I > 2σ(I)) | 0.1867 | 0.0824 |
| R_1_ (all data) | 0.0837 | 0.0580 |
| wR(F^2^) (all data) | 0.1934 | 0.0945 |
| Goodness-of-fit | 1.134 | 1.027 |
| CCDC deposition number | 2481483 | 2481482 |

**Table S2**. Single-crystal X-ray diffraction data for 2(**C_6_H_3_Cl_4_N**)·(**2,2-BPE**) and 2(**C_6_H_4_Cl_3_N**)·(**2,2-BPE**) at 290 K.

| compound name | 2(**C_6_H_3_Cl_4_N**)·(**2,2-BPE**) | 2(**C_6_H_4_Cl_3_N**)·(**2,2-BPE**) |
| --- | --- | --- |
| chemical formula | C_24_H_16_Cl_8_N_4_ | C_24_H_18_N_4_Cl_6_ |
| formula mass | 644.01 | 575.12 |
| crystal system | Monoclinic | Triclinic |
| space group | *P*2_1_/*n* | *P*ī |
| *a* (Å) | 15.6966(14) | 3.8748(4) |
| *b* (Å) | 3.8918(4) | 11.3132(10) |
| *c* (Å) | 21.976(2) | 14.2906(11) |
| α (˚) | 90 | 96.292(3) |
| β (˚) | 101.354(3) | 96.805(3) |
| γ (˚) | 90 | 90.904(3) |
| V (Å^3^) | 1316.2(2) | 618.03(10) |
| ρ_calc_ (g cm^-3^) | 1.625 | 1.545 |
| T (K) | 290 | 290 |
| Z | 2 | 1 |
| radiation type | MoKα (λ = 0.71073) | MoKα (λ = 0.71073) |
| absorption coefficient, μ/mm^-1^ | 0.880 | 0.717 |
| no. of reflections measured | 24603 | 23934 |
| no. of independent reflections | 3274 | 3061 |
| R_int_ | 0.0394 | 0.0488 |
| R_1_ (I > 2σ(I)) | 0.0307 | 0.0420 |
| wR(F^2^) (I > 2σ(I)) | 0.0773 | 0.1129 |
| R_1_ (all data) | 0.0414 | 0.0527 |
| wR(F^2^) (all data) | 0.0837 | 0.1200 |
| Goodness-of-fit | 1.031 | 1.052 |
| CCDC deposition number | 2481481 | 2481480 |

**2. ^1^H NMR Spectroscopic Data**


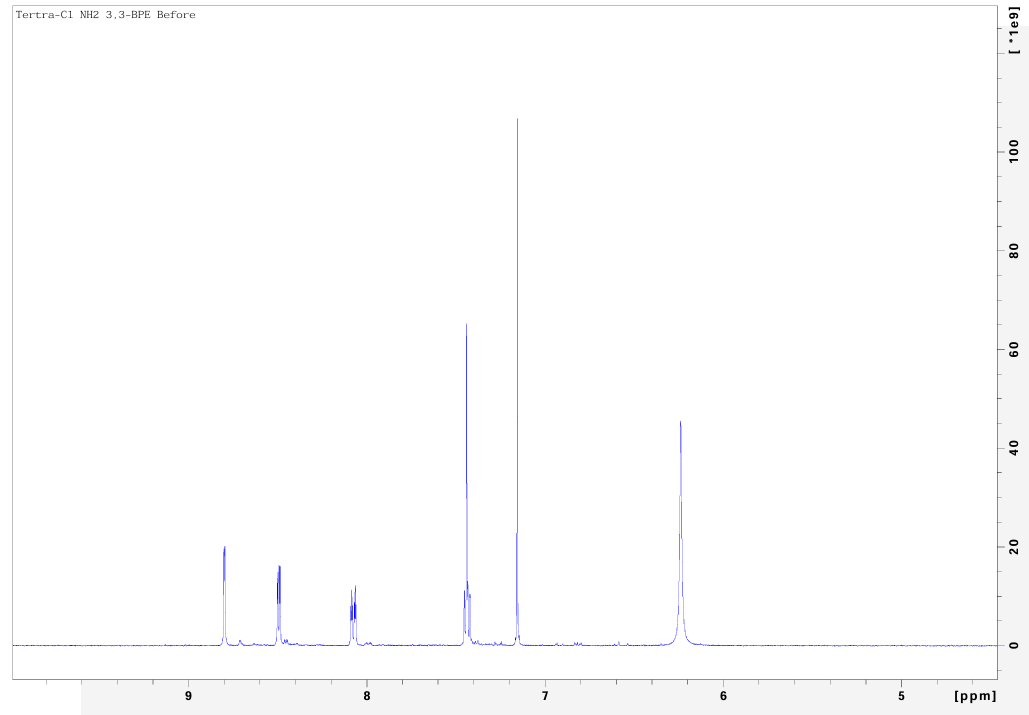


c

g

a

f

e

dc

b

a

g

b

d

c

f

e

**Figure S1: ^1^**H NMR spectrum of the co-crystal 2(**C_6_H_3_Cl_4_N**)·(**3,3-BPE**) before UV irradiation (400 MHz, DMSO-*d*_6_).

a

a

b

f’

e’

d’

g’

c’

b

g’

c’

d’

e’

f’

**Figure S2: ^1^**H NMR spectrum of the co-crystal 2(**C_6_H_3_Cl_4_N**)·(**3,3-TPCB**) after 60 hours of UV irradiation reaching a yield of 98% for the [2 + 2] cycloaddition reaction (400 MHz, DMSO-*d*_6_).


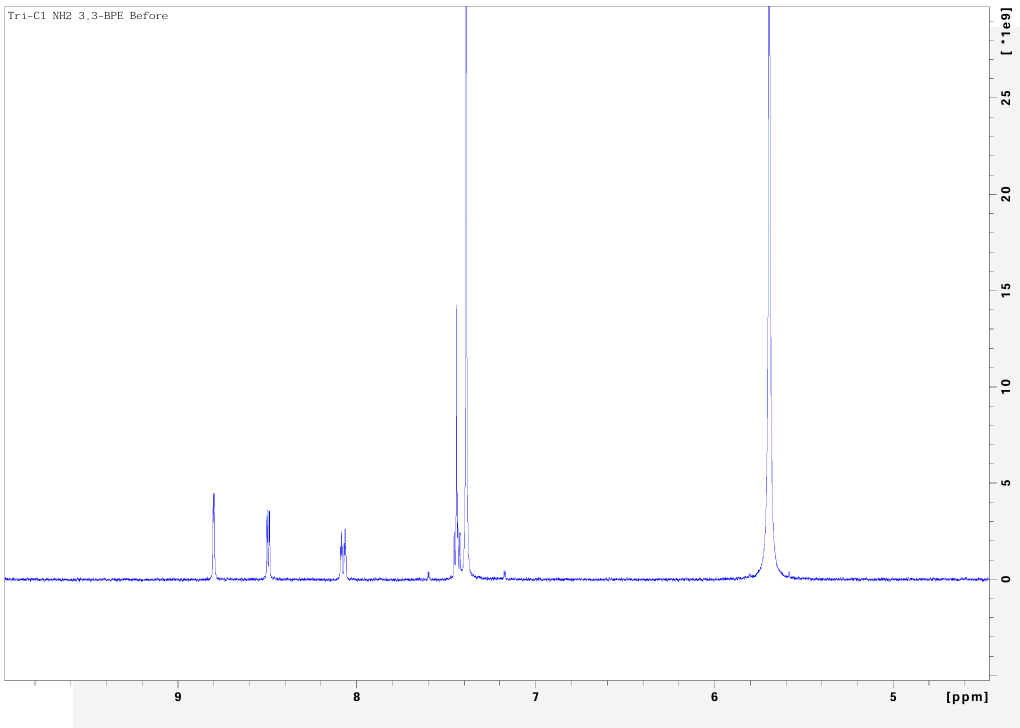


b

a

**
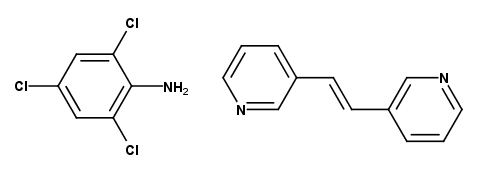
**

a

c

b

e

f

g

d

g

d

c

e

f

**Figure S3: ^1^**H NMR spectrum of the co-crystal 2(**C_6_H_4_Cl_3_N**)·(**3,3-BPE**) before UV irradiation (400 MHz, DMSO-*d*_6_).

a

**
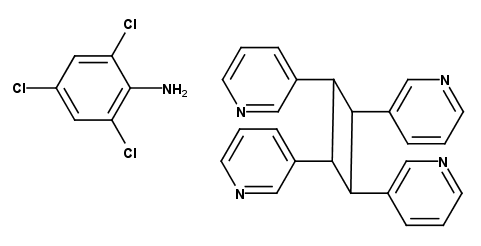
**

b

a

e’

f’

d’

c’

g’

g’

b

d’

c’

f’

e’

**Figure S4: ^1^**H NMR spectrum of the co-crystal 2(**C_6_H_4_Cl_3_N**)·(**3,3-TPCB**) after 16 hours of UV irradiation reaching a yield of 100% for the [2 + 2] cycloaddition reaction (400 MHz, DMSO-*d*_6_).


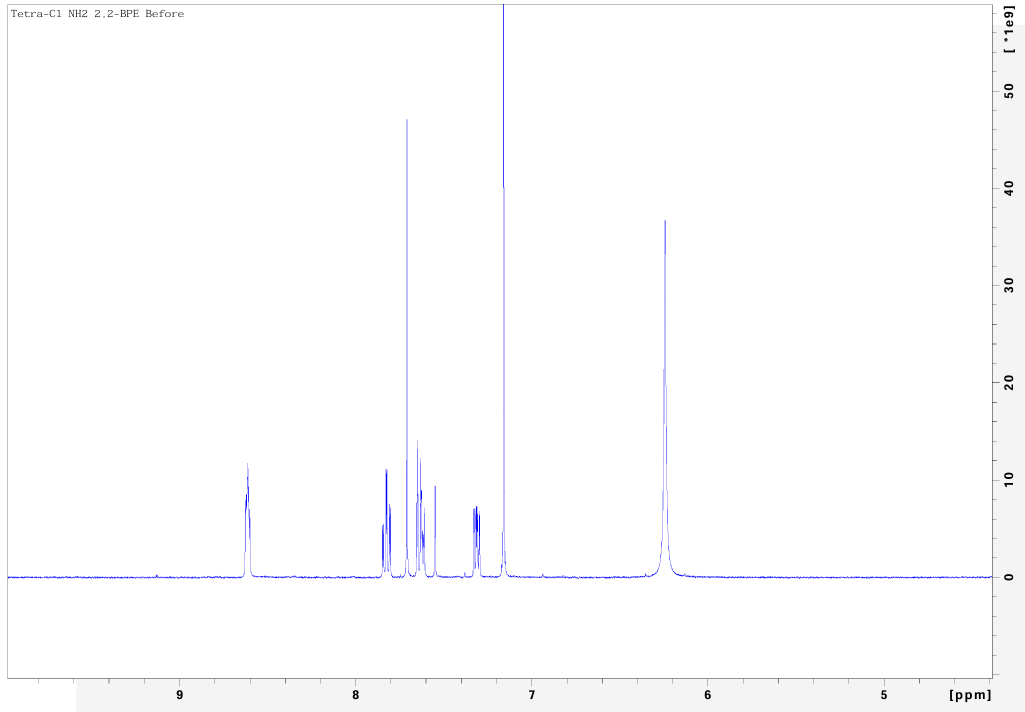
**
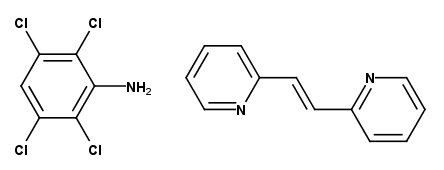
**

g

a

e

f

a

b

dc

c

g

b

f

e

c

d

**Figure S5: ^1^**H NMR spectrum of the co-crystal 2(**C_6_H_3_Cl_4_N**)·(**2,2-BPE**) before UV irradiation (400 MHz, DMSO-*d*_6_).

**
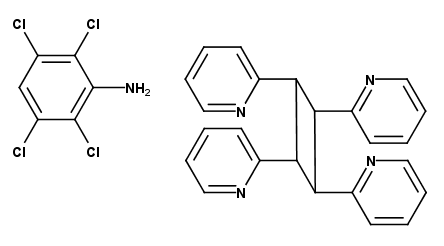
**

b

a

f’

e’

a

d’

g’

c’

g’

b

f’

d’

e’

c’

**Figure S6: ^1^**H NMR spectrum of the co-crystal 2(**C_6_H_3_Cl_4_N**)·(**2,2-TPCB**) after 40 hours of UV irradiation reaching a yield of 100% for the [2 + 2] cycloaddition reaction (400 MHz, DMSO-*d*_6_).

g0

g

f

e

a

b

d

c

a

f

e

c

d

b

**Figure S7: ^1^**H NMR spectrum of the co-crystal 2(**C_6_H_4_Cl_3_N**)·(**2,2-BPE**) before UV irradiation (400 MHz, DMSO-*d*_6_).

a

**
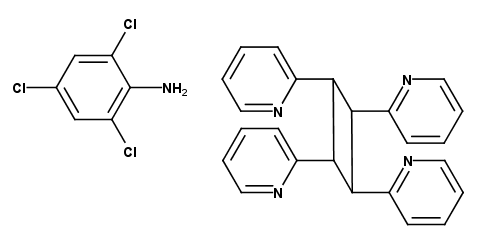
**

a

b

f’

e’

d'

g’

c'

g’

b

d'

f'

c'

e'

**Figure S8: ^1^**H NMR spectrum of the co-crystal 2(**C_6_H_4_Cl_3_N**)·(**2,2-TPCB**) after 60 hours of UV irradiation reaching a yield of 100% for the [2 + 2] cycloaddition reaction (400 MHz, DMSO-*d*_6_).

**3. Powder X-ray Diffraction Data
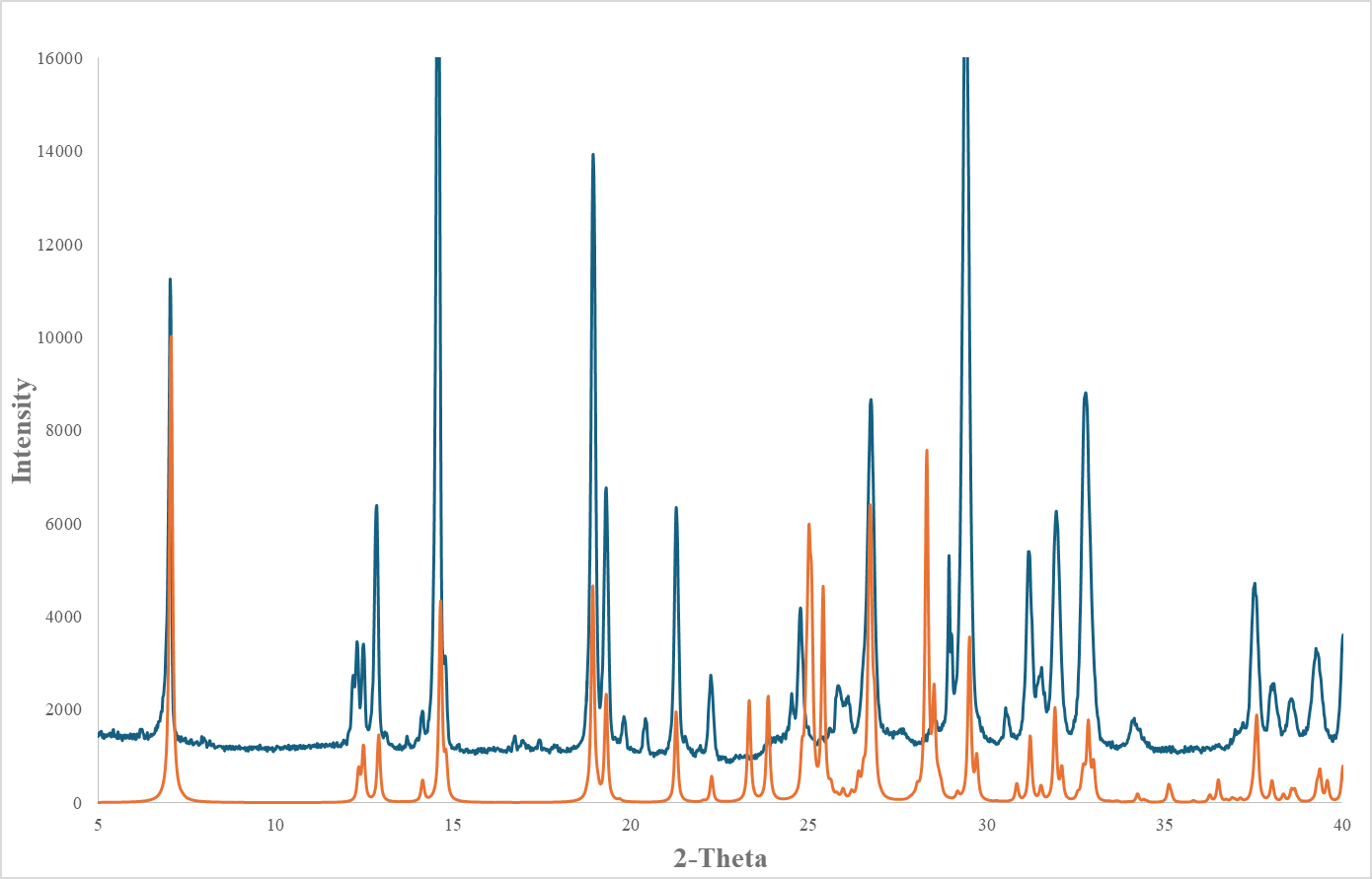
**

**Figure S9.** Powder X-ray diffraction data for the bulk sample that contains 2(**C_6_H_3_Cl_4_N**)·(**3,3-BPE**) (blue) along with the theoretical pattern for 2(**C_6_H_3_Cl_4_N**)·(**3,3-BPE**) (orange).

**
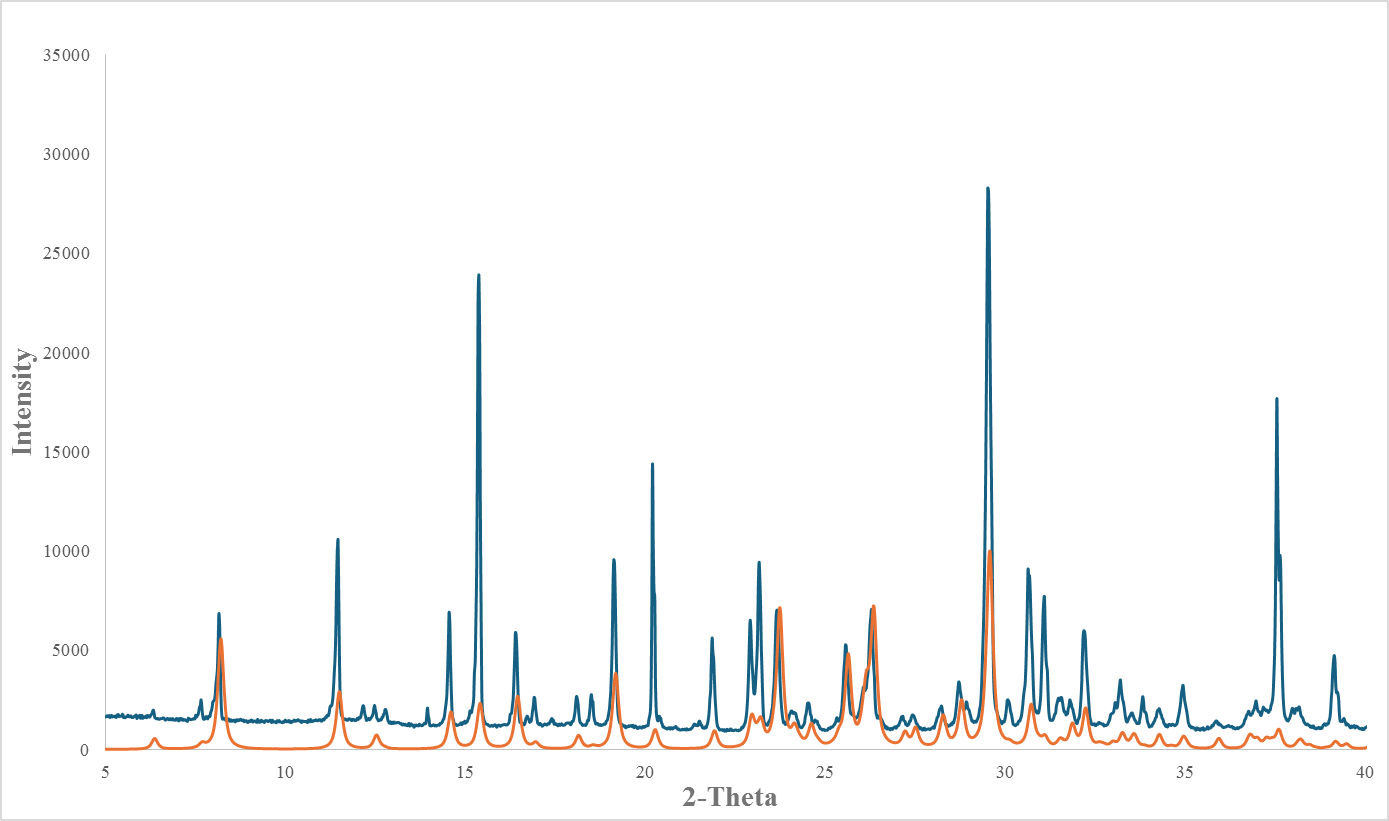
**

**Figure S10.** Powder X-ray diffraction data for the bulk sample that contains 2(**C_6_H_3_Cl_4_N**)·(**2,2-BPE**) (blue) along with the theoretical pattern for 2(**C_6_H_3_Cl_4_N**)·(**2,2-BPE**) (orange).

**
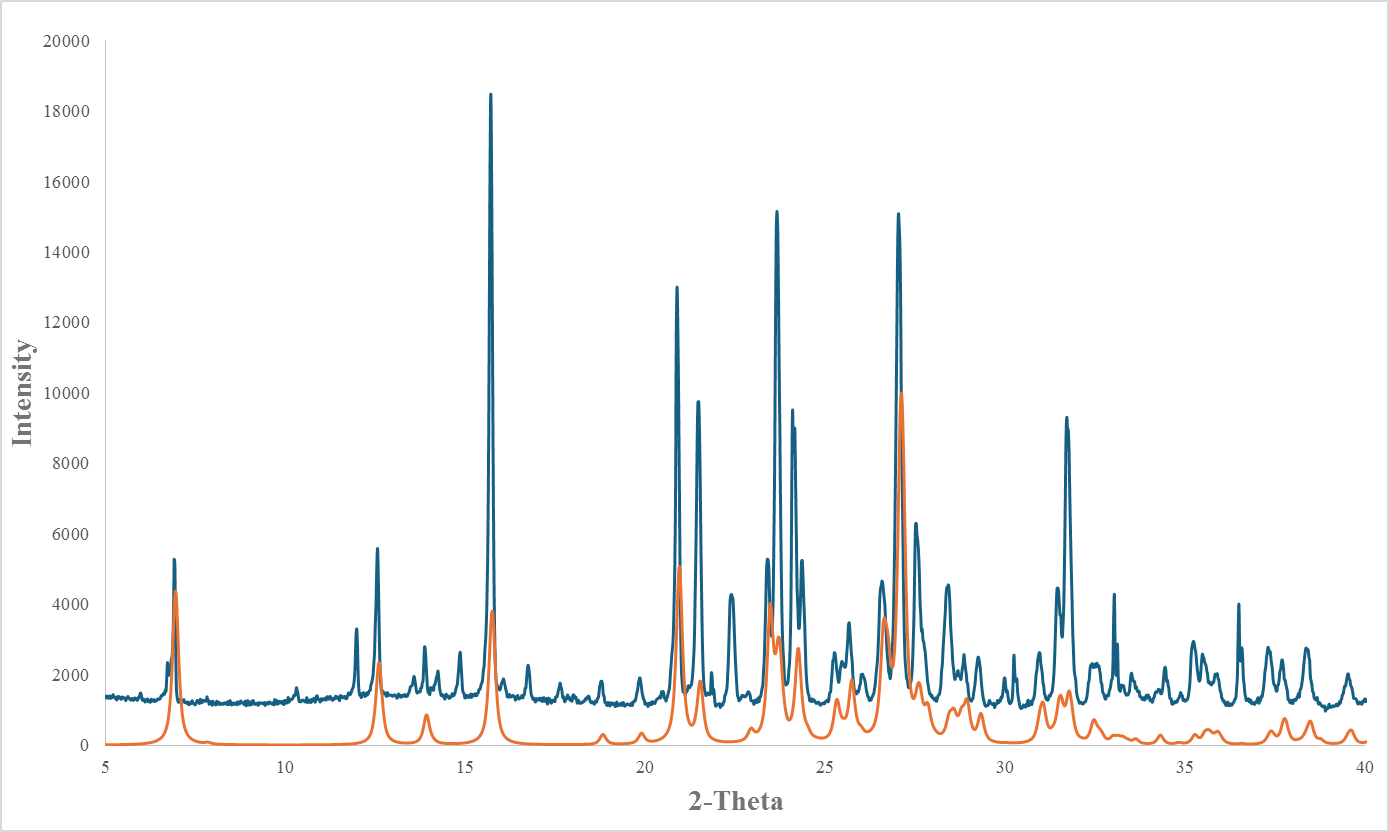
**

**Figure S11.** Powder X-ray diffraction data for the bulk sample that contains 2(**C_6_H_4_Cl_3_N**)·(**3,3-BPE**) (blue) along with the theoretical pattern for 2(**C_6_H_4_Cl_3_N**)·(**3,3-BPE**) (orange).

**
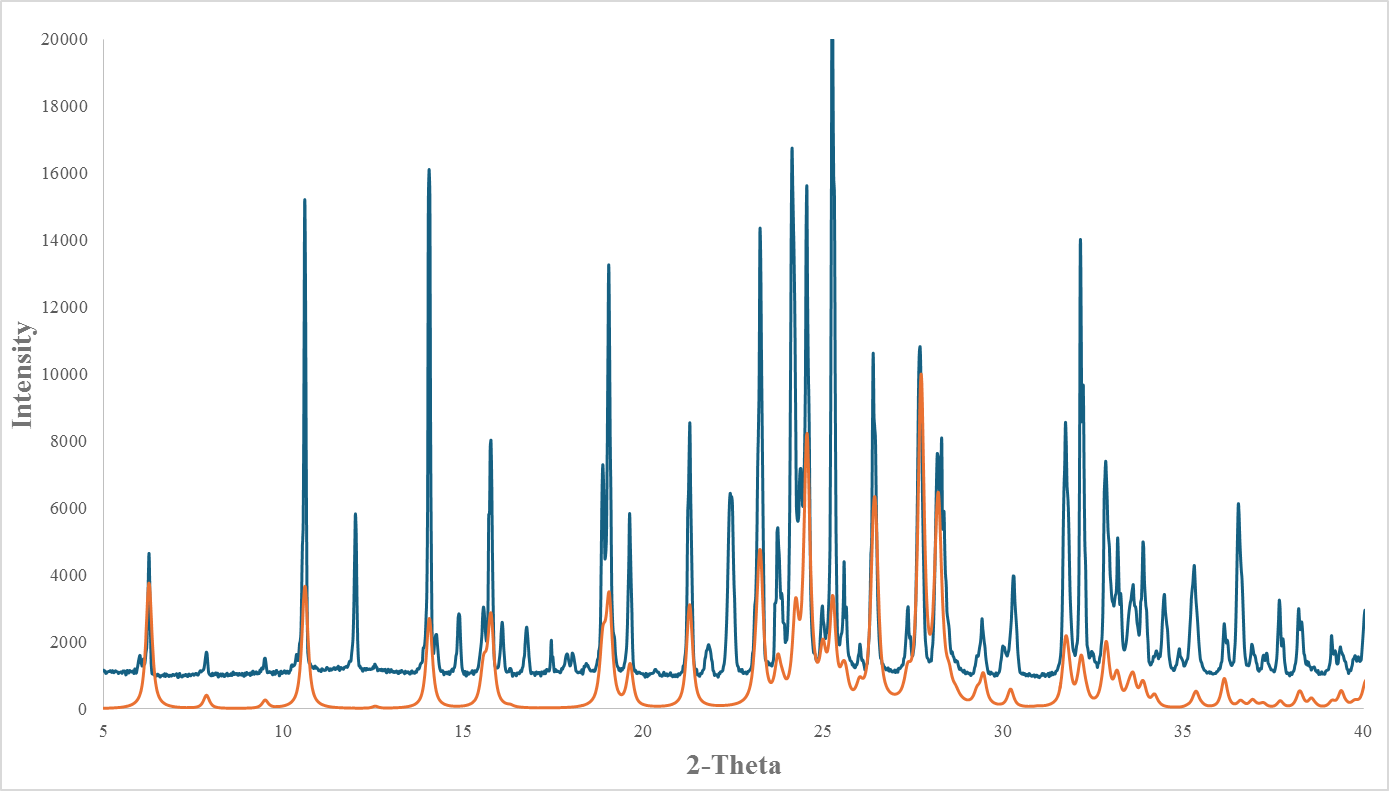
**

**Figure S12.** Powder X-ray diffraction data for the bulk sample that contains 2(**C_6_H_4_Cl_3_N**)·(**2,2-BPE**) (blue) along with the theoretical pattern for 2(**C_6_H_4_Cl_3_N**)·(**2,2-BPE**) (orange).

**References**

1. Bruker (2021). *APEX6*, *SAINT* and *SADABS*. Bruker AXS Inc., Madison, Wisconsin, USA.

2. G. M. Sheldrick, *Acta Crystallogr*., 2015, A**71**, 3-8.

3. G. M. Sheldrick, *Acta Crystallogr*., 2015, C**71**, 3-8.

4. O. V. Dolomanov, L. J. Bourhis, R. J. Gildea, J. A. K. Howard, H. Puschmann, *J. Appl. Cryst*., 2009, **42**, 339-341.
